# The Humoral Response to SARS-CoV-2 Vaccine in Hemodialysis Patients Is Correlated with Nutritional Status

**DOI:** 10.3390/vaccines11071141

**Published:** 2023-06-24

**Authors:** Merav Jacobson-Naftali, Odile Azoulay, Sigal Frishman, Lihi Godny, Boris Zingerman, Benaya Rozen-Zvi, Timna Agur

**Affiliations:** 1 Nephrology & Hypertension Department, Rabin Medical Center, Petaḥ Tikva 4941492, Israel; meravja@clalit.org.il (M.J.-N.); odila@clalit.org.il (O.A.); borisz@clalit.org.il (B.Z.); bnaiar@clalit.org.il (B.R.-Z.); 2 Nutrition Unit, Nephrology & Hypertension Department, Rabin Medical Center, Petaḥ Tikva 4941492, Israel; fsigal@clalit.org.il (S.F.); lihigo@clalit.org.il (L.G.); 3 Sackler Faculty of Medicine, Tel Aviv University, Tel Aviv 6997801, Israel

**Keywords:** hemodialysis patients, COVID-19 vaccine, antibody response, nutritional status, malnutrition, MIS

## Abstract

Hemodialysis patients are highly susceptible to poor nutritional status. Our objective was to investigate whether poor nutritional status during mRNA-SARS-CoV-2 vaccination is correlated with impaired vaccine responses. This retrospective study was conducted in two hospital-based dialysis units. The nutritional status of hemodialysis patients was assessed, using a malnutrition inflammation score (MIS) at the time of their first BNT162b2 vaccine dose. One month after the second vaccine dose, we performed a quantitative assessment of antibodies against the spike protein (anti-S1 IgG). A total of 115 hemodialysis patients, with an average age of 72 were enrolled in the study. Among them, 39 (33.9%) were female, and 67 (58.2%) had diabetes mellitus. In 43/115 (37.4%) patients, moderate to severe malnutrition (MIS > 5) was detected. Comparatively, malnourished patients showed a lower log-transformed mean level of anti-S1 IgG compared to those with normal nutrition (2.91 ± 0.83 vs. 3.25 ± 0.72, respectively, *p* = 0.024). In a multivariable analysis that adjusted for age, sex, and KT/V, the nutritional status assessed by an MIS remained inversely associated with an anti-S1 IgG response [B; −0.066 (−0.117 to −0.015)]. In conclusion, moderate to severe malnutrition in hemodialysis patients is associated with reduced humoral responses to BNT162b2 vaccination.

## 1. Introduction

Patients receiving hemodialysis are substantially vulnerable to coronavirus disease 2019 (COVID-19) morbidity and mortality. Nevertheless, the effectiveness of the widespread mRNA-COVID-19 vaccine among hemodialysis patients remains a concern [1,2]. Poor nutritional status is prevalent in this population, while the impact of nutritional status on their immune responses to vaccination is still unknown [3].

Variability in vaccine immunogenicity can be attributed to various factors, including formulation, dosages, and specific host factors, such as pre-existing immunity, age, medication use, and comorbidities [4,5]. The humoral response of the host and the production of antibodies by plasma cells following an antigenic challenge are considered key mechanisms for vaccine-induced protection. Additionally, T cells play a crucial role in vaccine-induced immunity by providing support to B cells, facilitating their maturation and antibody production. However, the limited evidence regarding the role of T cells in vaccine-induced protection is partly due to the challenges in accessing T cell responses [2,5].

Hemodialysis patients have consistently shown lower seroconversion rates, reduced antibody titers, and less sustained immune responses compared to healthy controls, following various vaccinations including those for HBV and influenza [2,4]. This diminished response is attributed to a chronic kidney disease (CKD)-induced premature ageing of the immune system and chronic systemic inflammation. Continuous oxidative stress, along with the dysfunction and apoptosis of immune cells, impairs both humoral and cellular immunity in these patients [2,6]. As a result, specific adjustments have been made to the routine vaccination regimens for dialysis patients, such as increased dosages or frequency, in order to compensate for their reduced responses to vaccination [4].

The recent COVID-19 pandemic has underscored the critical importance of implanting effective vaccination strategies to protect immunocompromised populations against infectious diseases [1,2,7]. Despite the high efficacy of the novel mRNA-SARS-CoV-2 vaccines in the general population, certain susceptible patient groups including those with hematological malignancies, solid organ transplant recipients, and HIV patients have presented significantly diminished immune responses to the standard vaccination regimen [8,9,10,11,12,13]. Similarly to other frail populations, hemodialysis patients display lower antibody titers, the rapid decay of antibodies, and reduced neutralizing antibody capacities, compared to age-matched controls [14,15,16,17,18]. Furthermore, patients receiving dialysis and those with myelofibrosis showed impaired memory B cell differentiation, as well as a decreased generation of vaccine-specific plasmablasts and mature memory B cells following two doses of the mRNA vaccination [10,19]. Fiorino et al. demonstrated that administrating a booster dose of the vaccine to patients with myelofibrosis, six months after the second dose, elicited a robust and significant increase in spike-specific B cell responses, akin to the responses observed in healthy controls [19]. However, given the heightened risk of COVID-19 infection among frail populations, including patients receiving hemodialysis, it is crucial to explore and adopt additional strategies to enhance vaccination response [2].

Hypoalbuminemia, defined as an albumin level below 3.5 g/dL, has been associated with a decreased immune response to the HBV vaccine in patients with CKD [3]. Consistent with these findings, our previous studies, as well as studies conducted by others, have demonstrated that hypoalbuminemia is an additional negative predictor of the immune response to BNT162b2 vaccination in hemodialysis patients [9,20,21,22,23]. Hypoalbuminemia is a major biomarker of malnutrition in vulnerable patient groups [24]. Malnutrition in frail patients is recognized as a critical health concern, associated with increased mortality and morbidity. The etiology of malnutrition is complex and multifactorial, with chronic disease and ageing being the primary causes in developed countries [25]. Dialysis patients, compared to other frail populations, face a heightened risk of malnutrition due to additional factors such as nutritional restrictions, impaired gastrointestinal absorption, an increased loss of micronutrients, and metabolic acidosis [24,26,27].The optimal functioning of the immune system relies on a wholesome and diverse nutrition profile, including sufficient protein, energy, vitamins, trace elements, and fibers [28]. Therefore, although further research is needed to confirm these findings, addressing and improving nutritional status, through dietary intervention, may potentially enhance vaccine immunogenicity [29,30].

Hypoalbuminemia can additionally arise from factors beyond nutritional status, including chronic inflammation and hypercatabolic states. Therefore, hypoalbuminemia alone cannot be relied upon as the sole indicator to determine nutritional status [30]. In dialysis patients, it is common to encounter both protein–energy wasting (PEW) and inflammation, which often coexist and can mutually influence each other. This phenomenon is referred to as the malnutrition–inflammation complex syndrome (MICS), as described by Kalantar-zadeh et al. [31]. To assess the nutritional status of dialysis patients, the malnutrition–inflammation score (MIS) has been proposed as a more comprehensive and reliable tool that incorporates the unique MICS phenomenon. The MIS includes medical history, dietary intake, physical assessment, body mass index (BMI), and laboratory results [32]. It is based on the widely used seven-point subjective global assessment (SGA); however, it additionally includes important nutritional markers such as BMI, serum albumin, and transferrin levels. The MIS has demonstrated a strong association with malnutrition-related morbidity, including hospitalizations and mortality, and thereby it has been recommended for use by the 2020 Kidney Disease/Dialysis Outcomes and Quality Initiative (KDOQI) Clinical Practice Guideline for Nutrition in Chronic Kidney Disease [26,30,31].

We aimed to utilize the comprehensive MIS tool to evaluate the association between nutritional status and humoral immunity following SARS-CoV-2 vaccination in hemodialysis patients.

## 2. Methods

### 2.1. Study Population and Design

This retrospective study was conducted in the two hospital-based dialysis units of the RMC (Rabin Medical Center). The study protocol was approved by the local Ethics Committee of the Rabin Medical Center, with the approval number 0823-21-RMC. The cohort included adult hemodialysis patients (age > 18 years), who received 2 doses of the BNT162b2 vaccine with a 21 days interval between December 2020 and February 2021. The vaccination program was carried out in accordance with the guidelines provided by national health authorities for out-of-hospital clinics. Patients who met the following criteria were excluded from the study: being on hemodialysis for less than 3 months, receiving immunosuppressive therapy, having received only 1 dose of the vaccine, being unable to provide informed consent, or having a prior, documented COVID-19 infection. Detailed demographic and clinical information and laboratory results were obtained from electronic medical records, including age, sex, comorbidities, use of immunosuppressive therapy, dialysis vintage, dialysis efficiency (KT/V), body mass index (BMI), dry weight, previous SARS-CoV-2 infection/s, COVID-19 vaccine dates, anti-S1 antibody level, and the most recent available laboratory results including albumin level, nPCR, serum transferrin level, serum ferritin level, lymphocyte count, and creatinine level.

### 2.2. Nutritional Status Assessments

The nutritional status of the patients was assessed using the MIS tool. The comprehensive MIS tool incorporates the evaluation of 10 measures, including the following: (1) loss of dry weight in the last 3–6 months; (2) appetite and nutritional intake; (3) gastrointestinal disorders; (4) daily function; (5) dialysis vintage and comorbidities; (6) physical examination for fat deposits; (7) physical examination of lean body mass; (8) body mass index (BMI); (9) serum albumin levels; and (10) serum transferrin levels.

Each parameter is assigned a score, ranging from 0 to 3. A score of 0 indicates optimal nutritional status, whereas a high score suggests poor nutritional status. The total sum of scores falls within the following ranges: 0 to 2 points: normal nutritional status; 3 to 5 points: nutritional status at risk, or mild malnutrition; above 5 points: moderate malnutrition; and above 8 points: severe malnutrition [32] (Supplementary Figure S1).

Based on their MIS scoring, patients were categorized into 2 groups: normal-nourished patients with an MIS ≤ 5 (ranging from normal to “at risk”) and malnourished patients with an MIS > 5 (ranging from moderate to severe) as described previously [32,33,34,35,36]. In the linear regression analysis, scores higher than 10 were adjusted to a maximum value of 10 for normalization purposes.

The assessment of the patients’ nutritional status was conducted around the time of the first vaccine dose administration. At our centers, comprehensive laboratory tests are performed monthly, and expert nephrology dietitians evaluate nutritional status every 3 months using the MIS tool. For those who missed the scheduled evaluation, a retrospective assessment was conducted based on detailed clinical evaluations and laboratory results from the medical records.

### 2.3. Antibody Response Assessment

Blood samples for anti-S1 IgG levels were collected during routine hemodialysis treatments, approximately 4 weeks after receiving the 2nd dose of the BNT162b2 vaccine. The samples were centrifuged to separate the serum and stored at −20 °C until analysis. A quantitative measurement of the IgG antibodies against the spike protein (anti-S1IgG) was performed, by using the SARS-CoV-2 IgG II Quant (Abbott©, Chicago, IL, USA) assay. The cutoff for positivity was 50 arbitrary units per ml (AU/mL) [37]. Based on the results of the World Health Organization International Standard study, the mathematical relationship of the Abbott unit (AU/mL) to the World Health Organization binding antibody units follows the subsequent equation: binding antibody units/mL = 0.142X AU/mL [37,38].

The primary outcome was defined as log-transformed IgG antibodies against the spike protein (Anti-S1-IgG);

### 2.4. Statistical Analyses

Categorical variables are presented as percentages, and continuous variables are presented as medians (interquartile ranges, IQRs) or means (±standard deviations, SDs), based on their distribution. Chi-square tests and repeated measure analyses of variance (ANOVA) were used to quantify the differences between groups in categorical variables and continuous variables, respectively.

A general linear regression model, with a confidence interval of 95% was conducted to determine the correlation between log-transformed antibody titers and MIS scores among hemodialysis patients. By using a multivariable linear regression analysis, we evaluated other factors associated with antibody response and assessed the strength and independence of nutritional status impact. All the variables were introduced into the multivariate analysis after testing for collinearity, using a forward stepwise regression model with a *p*-value below 0.05 used for inclusion. Predefined potential confounders, based on assumptions and literature were additionally forced into the model. The results presented as a change in the log-transformed antibody level, per unit of the explanatory variable (B coefficient).

Analyses were performed using IBM SPSS statistics, version 29.

## 3. Results

### 3.1. Patients’ Characteristics

**Figure 1:**
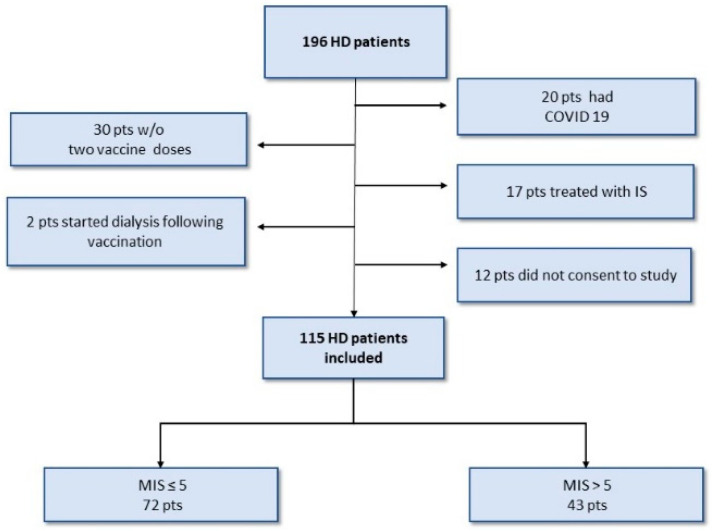
Patient flow chart.

A total of 196 dialysis patients were initially enrolled in this study. Among them, 20 had previously recovered from COVID-19 prior to vaccination, while 30 patients did not receive 2 vaccination doses during the study period. Additionally, 17 patients were undergoing treatment with immunosuppressive medications. Furthermore, 2 patients started dialysis after receiving their first vaccine dose, and 12 patients did not provide informed consent. Ultimately, the study cohort included 115 hemodialysis patients who met the inclusion and exclusion criteria (Figure 1). The patients’ characteristics are presented in Table 1. The average age of the participants was 72.2 ± 12.65 years. Out of the cohort, 39 (33.9%) were women, and 67 (58.2%) had diabetes mellitus. Among the cohort, 72/115 (62.6%) patients were classified as normal-nourished with an MIS score ≤ 5 and had a median MIS value of 3 (IQR, 0–5). Malnutrition, indicated by an MIS score > 5 was observed in 43 patients (37.4%), with a median MIS value of 7 (IQR, 6–22).

The malnourished group had an older mean age than the normal-nourished group (76.05 ± 10.65 vs. 69.9 ± 13.25, *p* = 0.011), with a lower mean serum albumin level (3.78 ± 0.34 mg/dL vs. 4.04 ± 0.22 mg/dL, *p* < 0.001), lower mean transferrin level (150.75 ± 25.21 mg/dL vs. 186.81 ± 39.71 mg/dL, *p* < 0.001), and reduced mean lymphocyte count (1.13 ± 0.57 k/micL vs. 1.38 ± 0.57 k/micL, *p* = 0.029). Both the mean dry weight and BMI were lower in the malnourished group, compared to the normal-nourished group (75.12 ± 17.70 vs. 67.39 ± 19.02, *p* = 0.038 and 27.52 ± 5.46 vs. 25.09 ± 5.10, *p* = 0.021, respectively) (Table 1).

### 3.2. Anti-S1 Antibody Response

The median anti-S1 IgG level in the malnourished group was 1151.5 AU/mL (IQR, 2.4–29,311.9), compared to 1848 AU/mL (IQR, 3.2–31,849.39) in the normal-nourished group.

**Figure 2:**
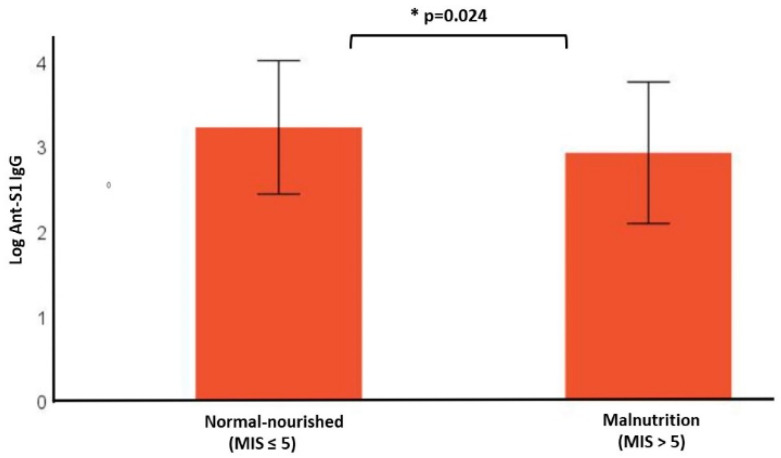
Antibody response among hemodialysis patients, according to nutritional status defined by malnutrition–inflammation score (MIS). The nutritional status of the patients was assessed, during the period of the first vaccine dose administration. Anti-S1 IgG level, was measured 1 month following 2nd dose of BNT162b2 vaccine. Results refer to the patient group with normal nutrition (*n* = 72) and patient group with malnutrition (*n* = 43). * (*p* < 0.05).

The mean log-transformed anti-S1 IgG was significantly lower in the malnourished group, compared to the normal-nourished group [3.25 ± 0.72 log AU/mL vs. 2.91 ± 0.83 log AU/mL, respectively, *p* = 0.024] (Table 2 and Figure 2).

### 3.3. A Linear Correlation between Antibody Response and Nutritional Status

**Figure 3:**
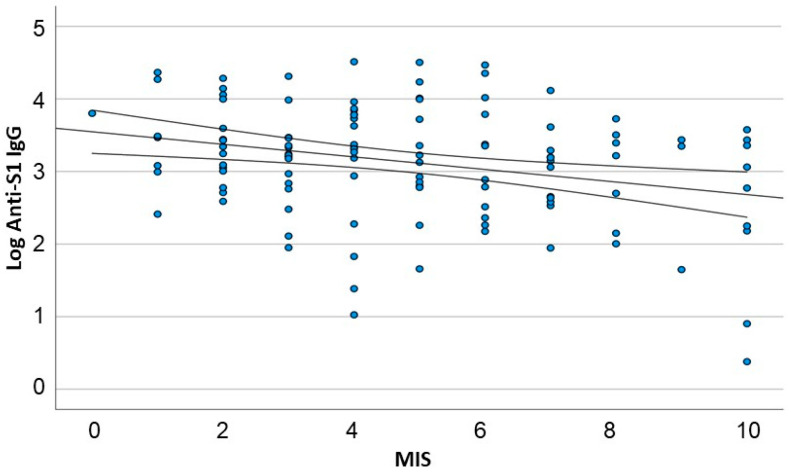
Linear regression of antibody response (log anti-S1 IgG), according to the MIS score. In the linear regression analysis, scores higher than 10 were normalized to 10 (*n* = 115).

This analysis revealed a significant inverse linear correlation between malnutrition, as measured by the MIS and log-transformed anti-S1 IgG levels. The correlation coefficient (B) was −0.087, with a 95% CI (−0.141 to −0.032) (*p* = 0.012) (Figure 3).

### 3.4. A Multivariate Analysis of Predictors of S1-IgG Antibody Levels in Response to BNT162b

We further conducted a multivariable analysis to examine the association between malnutrition and antibody response, while adjusting for potential confounding factors.

In addition to malnutrition, several other variables that were found to be significantly associated with log-transformed anti-S1 antibody levels in multivariable analyses were age (B, −0.022 95% CI −0.032 to −0.012, *p* < 0.001), female sex (B, −0.446, 95% CI −0.722 to −0.171, *p* = 0.002), and dialysis session efficiency assessed by KT/V (B, 0.578 95% CI 0.140 to 1.01, *p* = 0.01). Lymphocyte blood count was found to be positively correlated with antibody response in univariate analysis (B, 0.026, 95% CI 0.020 to 0.511, *p* = 0.034). However, in the multivariate analysis, the correlation did not remain statistically significant (Table 3).

Upon adjustment of the above variables: age, sex, and KT/V in multivariable analysis, the association between malnutrition and antibody response remained independent (B, –0.066, 95% CI −0.117 to −0.015, *p* = 0.012) (Table 3).

## 4. Discussion

This in-depth study, involving 115 hemodialysis patients from 2 hospital-based dialysis units found moderate to severe malnutrition to be prevalent in 37.3% of the patients. This study demonstrated an independent association between malnutrition status and a reduced antibody response to the BNT162b2 vaccination, as confirmed through both univariate and multivariate analyses. Furthermore, age and dialysis efficiency emerged as additional independent predictors of the humoral response to the SARS-CoV-2 vaccine among hemodialysis patients. These findings emphasize the importance of considering nutritional status, along with other demographic and treatment-related factors, when evaluating vaccine response in susceptible patient populations.

According to this current study, moderate to severe malnutrition, defined as an MIS score above 5, was detected in 37.3% of patients and was more prevalent among older patients. This prevalence aligns with a previous multicenter study, conducted in Israel, which reported a high risk of malnutrition in nearly 50% of 378 in-center hemodialysis patients [39]. Additionally, a meta-analysis of 90 studies encompassing 16,434 patients on maintenance hemodialysis revealed a prevalence of 28 to 54% of protein–energy wasting (PEW). Different nutritional assessment tools, dialysis modality, and the origin of the patient population, all contribute to the wide reported variability [40]. In line with this current study, a recent multicenter study in Spain, utilizing an MIS score for a malnutrition assessment with a cut-off point of 5, reported a prevalence of 51.7%. This study additionally identified ageing as a significant risk factor of malnutrition [33]. The association between malnutrition and ageing is well known in both CKD patients and non-CKD patients, due to shared etiologic factors, such as comorbid conditions associated with cachexia, physical inactivity, and frailty [25,27]. Indeed, the “obesity paradox” has been recognized in both dialysis and elderly populations, in whom overweight might be protective, paradoxically resulting in a better outcome [31,41].

The malnourished group in this study had lower levels of serum albumin, transferrin, and lower BMI, which are additional laboratory measures incorporated in the MIS tool beyond the conventional SGA score parameters. The inclusion of these quantitative markers in the MIS tool has been shown to improve its predictive performance for hospitalizations and mortality, compared to the original SGA tool [32]. It is important to note that hypoalbuminemia and transferrin may not solely indicate poor nutritional status, however, can additionally reflect inflammation. Nevertheless, malnutrition and inflammation often coexist and exacerbate each other, contributing to the development of the malnutrition–inflammation complex (MICS). Although inflammation can influence these laboratory markers, approximately 80% of the MIS score is based on pure nutritional variables. Therefore, while the contribution of potential inflammatory markers is present, it remains modest in the overall scoring. By incorporating these three quantitative variables, the MIS tool appears to provide a more comprehensive assessment tool for the nutritional status of dialysis patients [31,32].

The malnourished group additionally had a lower total lymphocyte count, compared to the normal-nourished group. Lymphopenia is an important marker of immune system disturbance. In severe COVID-19 disease, the majority of the patients will develop lymphopenia, likely due to the cytotoxic effect of the virus on T cells and the propagation of the inflammatory response, which leads to lymphocyte apoptosis [42]. A low peripheral lymphocyte count is additionally associated with malnutrition. Along with serum albumin level, it contributes to the calculation of the prognostic nutrition index (PNI), a measure of nutritional and immune status in cancer patients [43]. Similarly, a recent study focusing on patients with early-stage COVID-19 found a high prevalence of malnutrition as well as a correlation between malnutrition and a low lymphocyte count [44].

This current study found that the malnourished group with MIS scores above 5 had significantly lower levels of anti-S1 IgG antibodies, after receiving the 2nd dose of the BNT162b vaccine. The nutritional status of hemodialysis patients was identified as an independent predictor of diminished antibody responses to the mRNA anti-COVID-19 vaccination, in both univariable and multivariable analyses. These findings are consistent with a recent cohort study involving 205 hemodialysis patients, which additionally observed a correlation between the anti-spike antibody response to a priming dose of an adenovirus-vectored vaccine (ChAdOx1 nCoV-19) and nutritional status determined by the CONUT score. Intriguingly, the vaccine-induced neutralizing antibodies followed a similar pattern across different CONUT score groups. However, as opposed to the comprehensive MIS scoring, the COUNT score is calculated solely from laboratory measures including serum albumin, total cholesterol, and total lymphocyte count. In the above-mentioned study, the SGA tool, which assesses nutrition based on subjective impressions, showed a correlation with humoral response by univariate analysis, however not in the multivariate analysis [45].

Several studies have reported reduced antibody responses after vaccination in malnourished children [46,47]. Additionally, undernourished patients have shown a diminishing and delayed response to tuberculin skin tests (TST) following BCG vaccination [48]. The available data suggest that malnutrition can impair both innate and adaptive immunity, leading to a decrease in circulating B cells, a shift from Th1-associated to Th2-associated immune responses, and a reduced responsiveness of lymphocytes [47]. Furthermore, there is strong evidence indicating that the nutritional status of patients plays a significant role in infection-related morbidity and mortality, including COVID-19 disease and other infections. The underlying immune dysfunction caused by PEW, as well as essential micronutrient deficiencies such as zinc, selenium, and vitamins may impair immunity defense against the virus [49,50,51]. Dietary interventions appear to mitigate PEW and improve short- and long-term outcomes in dialysis patients [24,30]. However, further research is needed to determine whether nutritional support can enhance vaccine responses in susceptible populations.

Age and KT/V were additionally independently associated with reduced antibody responses. Uremia leads to the premature aging of the immune system. This includes a decrease in thymic output at an early age, loss of T cell receptor (TCR) diversity, and expansion of terminally differentiated CD4+ and CD8+ T cells. These immune alterations hinder the generation of protective immunity, both during natural infections and after vaccination [2,6]. While both ageing and dialysis adequacy are likely contributing factors to the reduced immunogenicity of the COVID-19 vaccine among dialysis patients, the available data are still controversial [14,22].

In this current study, female sex was additionally found to be associated with an inferior antibody response. Owing to the sex steroid hormones’ beneficial effects on both adaptive and innate immunity, women appear to be immune-privileged. Thus, young women often present a better response to various types of vaccination compared to men. During the transition to menopause, the decline in estrogen levels may enhance immunosenescence-related alterations, posing a greater risk of infection for postmenopausal women [52]. In line with the paradoxical effect between men and women, the finding of lower antibody responses among women may be attributed to their higher average age in the malnourished group.

In this present study, diabetes mellitus, which is additionally known to be associated with impaired immunity and poorer outcomes, did not independently predict an inferior immune response in a model that included nutritional status [14,53,54,55]. This finding suggests that malnutrition itself plays a significant independent role in reducing immunity among dialysis patients, which may overshadow the potential effect of diabetes. Therefore, despite concerns regarding the glycemic burden of nutritional interventions for diabetic patients, such considerations might not be applicable to malnourished dialysis patients.

Several limitations should be noted in our study. It was conducted as a retrospective, single-center study, which limits the generalizability of the findings to other populations or settings. We assessed for anti-S1 IgG responses only. Full immune protection following vaccination typically involves both humoral responses and cell-mediated immune responses [2,5]. In hemodialysis patients, the anti-S1 IgG response was found to be highly correlated with both neutralizing antibodies and cellular responses following mRNA-COVID-19 vaccinations [18,56]. However, quantifying a cell-mediated response is difficult, and a significant variation in T cell responses was observed between age-matched groups [5,12]. Our findings are based on the results of hemodialysis patients who received the widely used and effective mRNABNT162b2 vaccine. Nevertheless, the impact of malnutrition on the immune response to other SARS-CoV-2 vaccines may differ. Another limitation is that the antibody response was assessed at only one time point, following the second vaccination dose. As with other frail subjects, hemodialysis patients show delayed antibody responses following vaccination, which may only be captured by repeated measurements over time [10,13]. Finally, nutritional status was assessed using the MIS tool, which is the most comprehensive assessment available for dialysis patients. While hypoalbuminemia may be a surrogate for inadequate protein intake or other conditions such as inflammation or comorbidity, it is just one of ten other components of the MIS tool. Furthermore, a growing body of evidence supports the effectiveness of nutritional interventions in improving hypoalbuminemia [30].

In conclusion, considering their high mortality rate from infections, especially COVID-19, improving the vaccination response among susceptible patient populations is crucial. The findings that moderate to severe malnutrition in hemodialysis patients is associated with reduced humoral responses to SARS-CoV-2 vaccines support the importance of assessing nutritional status before vaccination. Research should be conducted to evaluate the effectiveness of nutritional interventions in improving vaccination responses among hemodialysis patients.

## Figures and Tables

**Table 1:** Baseline characteristics of hemodialysis cohort, according to nutritional status defined by MIS.

Variable	All	Normal-Nourished ^a^ MIS ≤ 5	Malnutrition ^b^ MIS > 5	*p*-Value
**Number**	115	72 (62.6%)	43 (37.4)	
**MIS** median (IQR)	5 (0–22)	3 (0–5)	7 (6–22)	<0.001
**Recipient sex**				0.564
**Female sex** (**%**)	39 (33.9)	23 (31.9)	16 (37.2)	
**Male sex** (**%**)	76 (66.1)	49 (68.1)	27 (62.8)	
**Age** (**years**) mean ± SD	72.2 ± 12.65	69.9 ± 13.25	76.05 ± 10.65	0.011
**Diabetes Mellitus** (**%**)	67 (58.2)	41 (56.9)	26 (60.4)	0.711
**Albumin** (**g/dL**) mean ± SD	3.94 ± 0.30	4.04 ± 0.22	3.78 ± 0.34	<0.001
**Hemoglobin** (**g/dL**) mean ± SD	10.64 ± 1.16	10.68 ± 1.22	10.58 ± 1.07	0.657
**Lymphocyte count** (**K/micl**) mean ± SD	1.28 ± 0.58	1.38 ± 0.57	1.13 ± 0.57	0.029
**KT/V** mean ± SD	1.43 ± 0.31	1.39 ± 0.30	1.48 ± 0.32	0.185
**nPCR** (**gr/kg/d**)	1.1 ± 0.26	1.11 ± 0.25	1.08 ± 0.27	0.53
**Creatinine** (**mg/dL**) mean ± SD	6.91 ± 1.95	7.15 ± 1.86	6.52 ± 2.07	0.098
**Transferrin** (**mg/dL**) mean ± SD	39.21 ± 174.27	39.71 ± 186.81	25.21 ± 150.75	<0.001
**Ferritin** (**mg/dL**) mean ± SD	699.15 ± 459.96	442.66 ± 655.76	521.42 ± 781.44	0.236
**Dry weight** (**kg**) mean ± SD	72.3 ± 18.4	75.12 ± 17.70	67.39 ± 19.02	0.038
**BMI** (**kg/m^2^**) mean ± SD	26.63 ± 5.44	27.52 ± 5.46	25.09 ± 5.10	0.021
**Dialysis vintage** (**months**) mean ± SD	33.1 ± 40.89	37.85 ± 33.52	45.52 ± 32.29	0.245

^a^: normal-nourished patients = ranging from normal to at risk (MIS ≤ 5); ^b^: malnourished patients = ranging from moderate to severe malnutrition (MIS > 5). BMI, body mass index; nPCR, normalized protein catabolic rate; MIS, malnutrition–inflammation score; IQR, interquartile range; and SD, standard deviation.

**Table 2:** Humoral response according to nutritional status.

	All	Normal-Nourished MIS ≤ 5	Malnutrition MIS > 5	*p*-Value
**Patients number (%)**	115	72 (62.6%)	43 (37.3%)	
**Anti-S1 IgG** (AU/mL) (median [IQR])	1559.80 (2.4–31,849.3)	1848 (3.2–31,849.39)	1151.5 (2.4–29,311.9)	
**LOG Anti-S1 IgG** (mean ± SD)	3.12 ± 0.78	3.25 ± 0.72	2.91 ± 0.83	0.024

MIS, malnutrition–inflammation score; IQR, interquartile range; and SD, standard déviation.

**Table 3:** Multivariate analysis of predictors of S1-IgG antibody levels, in response to BNT162b2 vaccine among hemodialysis patients.

Variable	HD Patients N = 115	Univariate B (95% CI)	*p* Value	Multivariate B (95% CI)	*p* Value
**Age** (per year) mean ± SD	72.2 ± 12.6	−0.023	<0.01	−0.022 (−0.032 to −0.012)	<0.001
**Female sex** (%)	39 (33.9)	−0.365 (−0.66 to −0.067)	0.017	–0.446 (−0.722 to −0.171)	0.002
**Hemoglobin** (g/dL) mean ± SD	10.64 ± 1.15	−0.019 (−0.144 to −0.107)	0.766	-	
**Diabetes mellitus**	67 (58.2)	−0.079 (−0.372 to −0.214)	0.595	-	
**KT/V** mean ± SD	1.43 ± 0.30	0.208 (−0.271 to −0.688)	0.391	0.578 (0.140 to −1.01)	0.01
**nPCR** mean ± SD	1.1 ± 0.25	0.342 (−0.233 to −0.908)	0.233	–	–
**Lymphocyte count** (K/micl) mean ± SD	1.28 ± 0.58	0.265 (0.020 to −0.511)	0.34	–	–
**MIS** mean ± SD	4.8 ± 2.56	−0.087 (−0.141 to −0.032)	0.02	–0.066 (−0.117 to −0.015)	0.012

B > 0 indicates a positive correlation with the log antibody titer. nPCR, normalized protein catabolic rate; and MIS, malnutrition–inflammation score.

## Data Availability

Data from this study will be available upon request from the corresponding author.

## Supplementary Materials

The following supporting information can be downloaded at: https://www.mdpi.com/article/10.3390/vaccines11071141/s1, Figure S1: Components of the comprehensive MIS.

## Abbreviations

ANOVA: repeated measure analysis of variance; BMI: body mass index; CKD: chronic kidney disease; K/DOQI: Kidney Disease Outcomes Quality Initiative; MICS: malnutrition–inflammation complex syndrome; MIS: malnutrition–inflammation score; SGA: subjective global assessment; PEW: protein–energy wasting; nPCR: normalized protein catabolic rate; PNI: prognostic nutrition index; RMC: Rabin Medical Center; TCR: T cell receptor; and TST: tuberculin skin test.
