# Supplementary figures and images for: The Humoral Response to SARS-CoV-2 Vaccine in Hemodialysis Patients Is Correlated with Nutritional Status
Source: Vaccines (Basel). 2023 Jun 24;11(7):1141. doi: 10.3390/vaccines11071141 (PMC10386095; doi:10.3390/vaccines11071141)

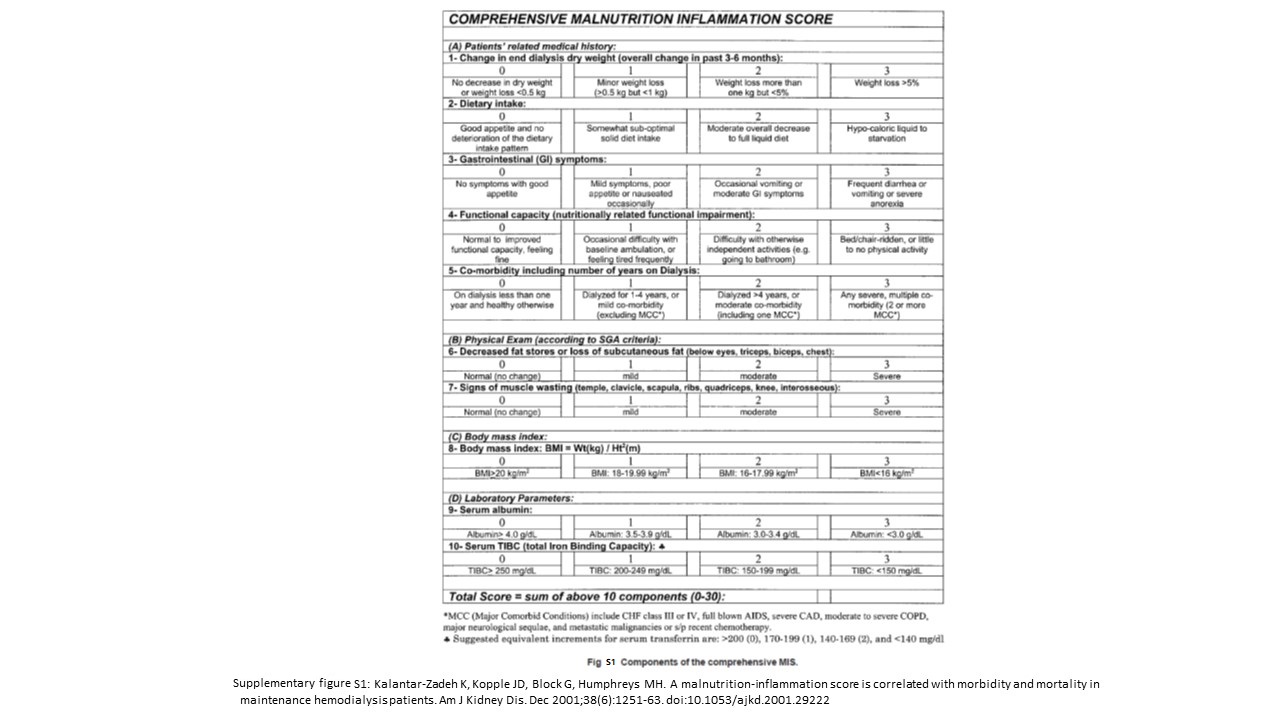

Supplement: Supplementary file 1 [file vaccines-11-01141-s001.zip › vaccines-2426369-supplementary.jpg]
